# Misplaced and Knotted Nasogastric Tubes in Infants and Children: Report of Two Cases

**DOI:** 10.14740/jmc5272

**Published:** 2026-02-02

**Authors:** Mitchell Hughes, Kortney Holland, Joseph D. Tobias

**Affiliations:** a Heritage College of Osteopathic Medicine - Dublin, Ohio and Ohio University, Athens, OH, USA; b Department of Anesthesiology & Pain Medicine, Nationwide Children's Hospital and The Ohio State University, Columbus, OH, USA

**Keywords:** Nasogastric tube, Knot, Endotracheal tube, Pediatric anesthesiology, Airway complication

## Abstract

Nasogastric (NG) tubes are frequently placed during intraoperative care to decompress the gastrointestinal tract and prevent postoperative complications in patients with gastrointestinal obstruction or ileus. Although placement during anesthetic care is generally straightforward, misplacement or knotting of the NG tube may occur. We present two cases in pediatric-aged patients where difficulties were noted with passing and/or removal of the NG tube. Techniques for placement of NG tubes are reviewed, previous reports of misplacement or difficulties with placement presented, and strategies to prevent these complications discussed.

## Introduction

As the name indicates, nasogastric (NG) tubes are inserted through the nares and into the stomach. NG tubes are typically used for decompression of the stomach in patients with gastrointestinal obstruction or ileus, to provide enteral nutrition and administer medications, or for various diagnostic purposes [1]. The first reports of NG tubes and similar devices appear in the literature in the late 19th to early 20th century. The Levin tube, developed by Dr. Abraham Levin in 1921, was a non-weighted, single-lumen rubber tube that had multiple distal perforations to facilitate NG aspiration, lavage, and gavage [2, 3]. The introduction and design of the Levin tube led to the evolution of standard NG tubes through innovations in materials and designs including development of the modern-day Salem Sump, a dual lumen tube, commonly used for NG decompression and medication delivery [4].

Although generally easy to place and free of significant long-term adverse effects, misplacement or knotting of the NG tube may occur. We emphasize the potential impact that these problems may impose in infants and children by presenting two pediatric-aged patients where difficulties were noted with passing and/or removal of the NG tube during intraoperative care. Techniques for placement of NG tubes are reviewed, previous reports of misplacement or difficulties with placement presented, and strategies to prevent these complications discussed. We specifically aim to demonstrate the potential for knot formation even when appropriate technique is used.

## Case Reports

Review of these case and presentation in this format followed the guidelines of the Institutional Review Board of Nationwide Children’s Hospital. This review was conducted in compliance with the ethical standards of the responsible institution on human subjects as well as with the Helsinki Declaration.

### Case 1

**Figure 1:**
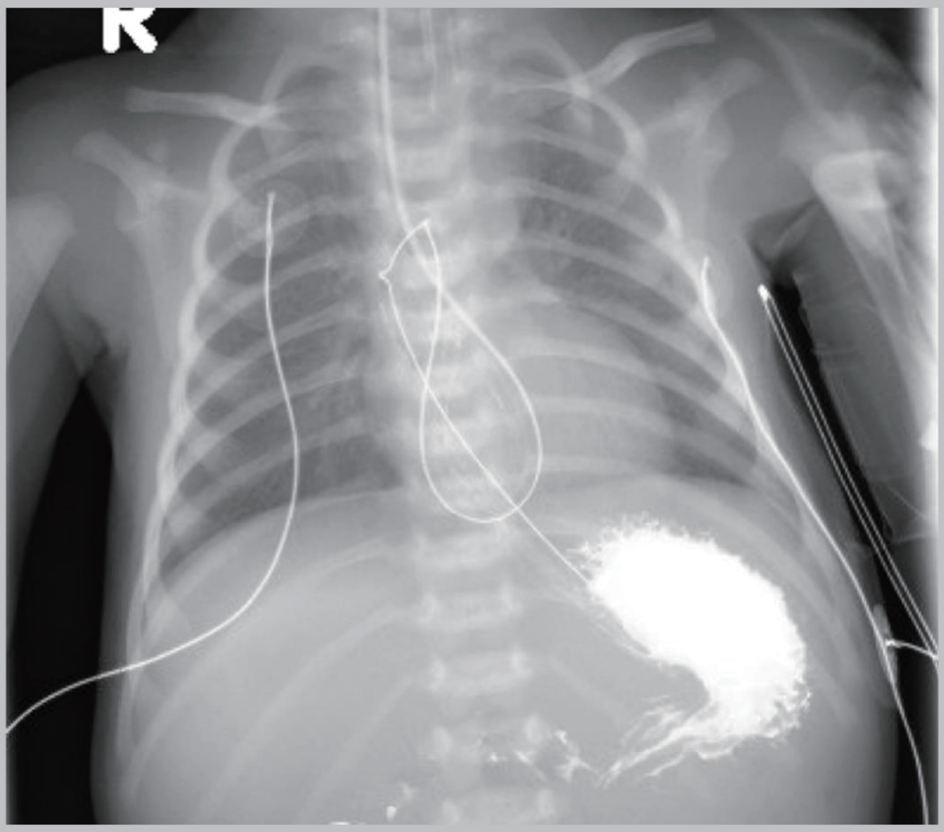
Initial intraoperative chest radiograph revealing looping of the nasogastric (NG) tube within the esophagus and its tip in the fundus of the stomach.

**Figure 2:**
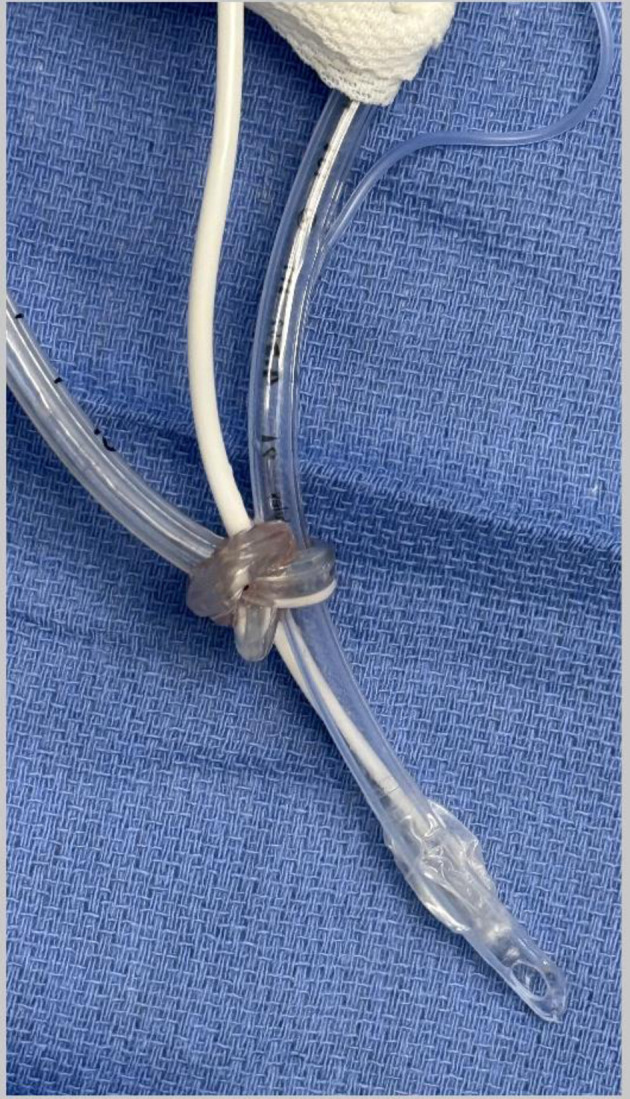
Nasogastric (NG) tube following removal showing the NG tube knotted around the endotracheal tube and the temperature probe.

The patient was a 6-year-old, 18.2 kg, girl with history of a Wilms tumor, status post right nephrectomy (recurrence), and right paracaval mass resection who presented to the emergency department (ED) in septic shock after developing acute abdominal pain over the past 12 h. The patient had received a dose of chemotherapy the day prior to her presentation to the ED. The patient was admitted to the pediatric intensive care unit (PICU) for resuscitative measures and workup of the acute abdominal pain with a differential diagnosis including volvulus versus other etiologies of small bowel obstruction with possible ischemic bowel. The patient was subsequently taken to the operating room (OR) for an exploratory laparotomy. There was no history of prior NG use or placement. Following the induction of general anesthesia and endotracheal intubation, an NG tube was placed using standard intraoperative procedure with gentle insertion through a nare to the predetermined length. No issues were noted with placement, but during abdominal exploration its tip could not be felt within the stomach by the surgery team. An intraoperative radiograph demonstrated looping of the NG within the esophagus and its tip high in the fundus of the stomach (Fig. 1). During attempts to adjust the position of the NG tube intraoperatively, the endotracheal tube (ETT) became dislodged and there was loss of end-tidal carbon dioxide (ETCO_2_) waveform. The NG tube, ETT, and esophageal temperature probe were removed and the trachea was immediately reintubated without difficulty with a new ETT. Upon further evaluation of the ETT and NG, it was noted that the NG was knotted around the ETT and the temperature probe (Fig. 2), so that manipulation of the NG tube (withdrawal) resulted in inadvertent tracheal extubation. Following reintubation of the trachea, the patient tolerated the remainder of the procedure well and upon completion of the procedure was returned to the PICU.

### Case 2

**Figure 3:**
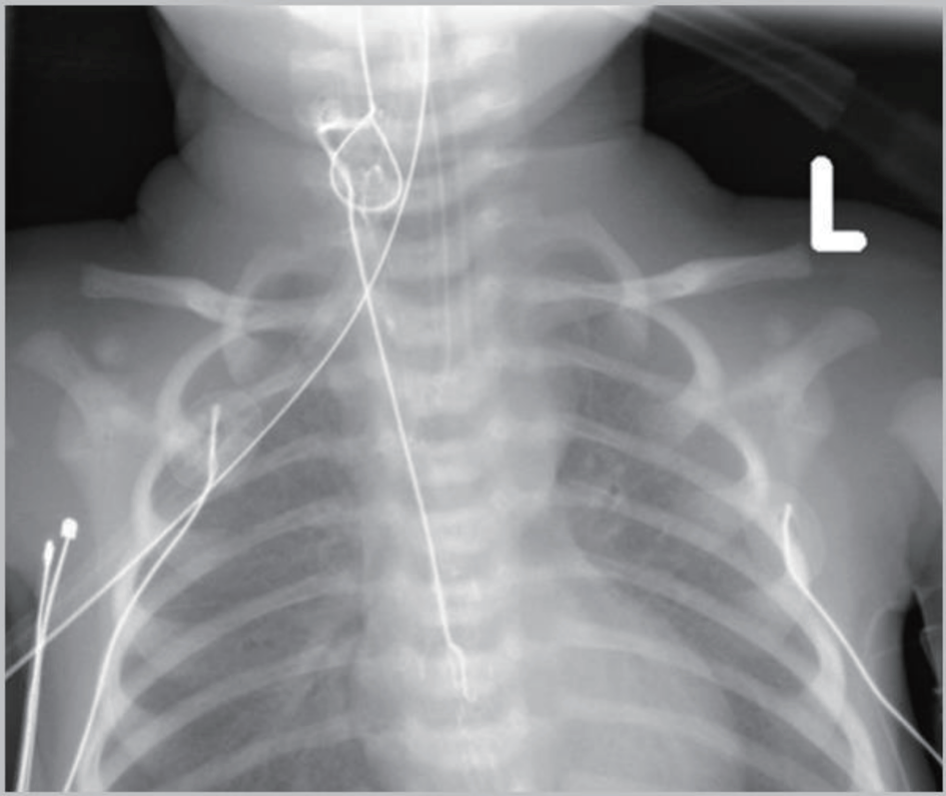
Postoperative chest radiograph obtained after attempted withdrawal of the nasogastric (NG) tube showing the NG with a knot in the proximal esophagus.

**Figure 4:**
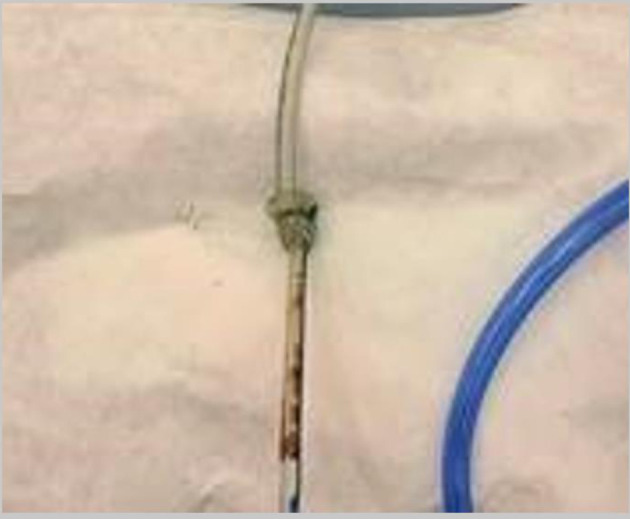
Knotted nasogastric tube after removal by the pediatric otorhinolaryngologist.

The patient was a 2-day-old, 2.12 kg, premature female infant with history of bilious emesis who presented with duodenal atresia. The patient was taken to the OR for an exploratory laparotomy with plans for a duodenoduodenostomy. Following the induction of general anesthesia and endotracheal intubation, an NG tube was placed for intraoperative decompression of the GI tract. The procedure was tolerated well, and intraoperatively duodenal atresia was confirmed. An intraoperative radiograph was not obtained to confirm NG position. Postoperatively, while the patient was recovering in the NICU, a chest radiograph showed a knot in the NG tube which was positioned in the distal esophagus (Fig. 3). The NG tube could not be removed at the bed side as there was resistance to its withdrawal. Given the newly created duodenoduodenostomy and concern for possible esophageal injury, the patient was returned to the OR, so that the NG tube could be removed under direct visualization and if needed, the esophagus inspected following removal. Following the induction of general anesthesia with an endotracheal tube already in place, direct laryngoscopy was performed by the pediatric otorhinolaryngology service, and the NG tube was removed under direct vision (Fig. 4). The esophagus was not injured. A new NG tube was replaced under direct vision. The patient tolerated the procedure well and upon completion of the procedure was returned to the NICU.

## Discussion

Insertion of an NG tube is a commonly performed procedure in hospitalized patients for gastric decompression during or following abdominal surgery, enteral nutrition, and medication administration. Despite its frequent use and advancements in its design over the years, placement and subsequent use are not without the potential for adverse effects or complications as noted in our two patients. Previous reports in both children and adults have demonstrated that even with appropriate insertion techniques, knots may form as the NG tube is advanced into place [5–7].

In general practice, placement of NG tubes is performed blindly, without instrumentation, through the nares into the stomach. The depth of placement to ensure correct intragastric location can be estimated using the nose, ear, mid-umbilicus (NEMU) method as described originally at The Royal Children’s Hospital Melbourne [8]. This distance is determined by measuring from the tip of the nose to the earlobe, and then to the middle of the abdomen, halfway between the breastbone and the umbilicus. The tube is then marked with the estimated length and inserted through the nare. Although this is generally a useful method for estimating the depth of placement, insertion and removal of NG tubes can have a wide variety of complications.

The most common complications with NG tube placement include patient discomfort or epistaxis during placement and sinusitis with long-term use. However, more severe complications have been reported including trauma to the nasopharynx with development of a blind passage, laryngeal injury, or perforation of the esophagus or stomach with bleeding or ulceration. Additionally, during passage, the distal portion of the NG tube is susceptible to kinking, coiling, or even formation of a knot as noted in our patients [9]. Additionally, inadvertent entry into the trachea, lungs, or brain, can lead to pneumothorax, aspiration, pneumonia, perforation, introduction of medications into the wrong space, or even death [10].

Prior reports suggest that the risk of complications is greater in patients with altered consciousness, heavy sedation, and in the presence of critical illness. Without maneuvers to facilitate NG passage, the failure rate can be as high as 50% on the first attempt in patients who are anesthetized, paralyzed, or tracheally intubated [9, 10]. In a prospective study of 60 patients who required NG tube placement, patients were randomized to a neutral or flexed head position [11]. NG tube placement was successful on the first attempt in 80% of the patients with flexion of the head compared to 50% with the head in a neutral position. In addition, patients with an ETT or tracheostomy in place have been reported to have an increased risk of complications with NG tube placement. In a prospective study of 740 NG tube insertions in the ICU, 13 of 14 patients with misplacement of the NG tube, had an ETT in place [12].

Our two cases highlight the potential for unique challenges during the insertion of an NG tube in a tracheally intubated and anesthetized patient, demonstrating the need for vigilance during insertion, repositioning, or removal of the NG. These two cases demonstrate the potential for coiling and/or knotting of the NG tube. Risk factors for knotting of an NG tube are outlined in Table 1. Of the factors listed, use of narrow bore tubes and excessive tube length allowing self-knotting within the stomach are the most frequently reported predisposing factors. In our first case, the NG tube coiled and knotted around the ETT and temperature probe resulting in inadvertent tracheal extubation during NG tube manipulation. Fortunately, this was quickly identified by the loss of effective tidal volume and the ETCO_2_ waveform and the ETT was safely replaced without patient harm. Following replacement of the ETT, direct visualization identified the knot of the NG tube that was around the ETT and the temperature probe. In our second case, the knot in the NG tube caused difficulties in withdrawing the tube from the esophagus, necessitating return to OR for direct visualization and removal. Direct laryngoscopy was ultimately performed to remove the NG tube. This case demonstrates the need for vigilance during removal of the device and an option for alternative techniques when resistance is felt during withdrawal of the NG tube (Table 2).

**Table 1:** Risk Factors for NG Tube Knot Formation

Use of narrow bore tubes
Excessive tube length in the stomach
Gastric anatomy including small stomach volumes
Altered gastric anatomy following surgery
Vigorous peristalsis activity
Prolonged duration of tube placement
Excessive tube manipulation or movement
Rapid insertion or withdrawal of the ETT

ETT: endotracheal tube; NG: nasogastric.

**Table 2:** Pathway for NG Removal When Resistance Is Encountered

1. High index of suspicion when excessive resistance is noted on attempted NG tube removal.
2. Attempts at withdrawal should stop—never pull against resistance due to risk of esophageal or airway injury.
3. Imaging studies (lateral/AP neck or skull radiograph) may identify the knot and its position.
4. Attempt to visualize the knot in the oropharynx. If it can be seen, it may be possible to hold the knot with a forceps and cut above it.
5. The knot is then removed through the mouth with the distal end of the NG while the remainder of the NG tube (proximal end) is removed through the nare.
6. If the knot is more distal, removal under direct vision with sedation/anesthesia may be required. This may require consultation with otolaryngology or gastroenterology.

AP: anteroposterior; NG: nasogastric.

Previous clinical work has demonstrated that the “at-risk patient population” for complications of NG tube placement and removal are those who are tracheally intubated, have altered mental status, or are unable to protect their airway. Special precautions in these patients as outlined by the report of Hanna et al may be needed to ensure proper placement and removal of NG tubes [13]. Furthermore, radiographic confirmation is suggested to document that the tip of the NG tube is correctly located within the stomach prior to its use for medication or nutrition administration. More recently, the use of bedside point-of-care ultrasound (POCUS) has been suggested as technique to not only guide and facilitate NG placement, but also confirm that the tip is in the correct location [14].

Modifications have been proposed to ease insertion of NG tubes, such as lateral neck pressure, head flexion, reverse Sellick’s maneuver, or freezing the NG tube itself [9]. Reverse Sellick’s maneuver involves lifting the cricoid cartilage forward to open the esophagus and widen the entrance, while the frozen technique involves strengthening the distal end by freezing it to increase rigidity for proper guidance in positioning. A study of 195 anesthetized, tracheally intubated adult patients who required NG tube insertion for abdominal surgery found that successful placement was the highest using the reverse Sellick’s maneuver, followed by freezing the tube, and lastly by blind insertion [12]. Of note, bleeding, coiling, and kinking of the NG tube is more likely with freezing the tube. The heightened rigidity leads to a higher success rate in placement due to better guidance throughout the GI tract, but comes with increased risk of trauma, bleeding, or kinking. Thus, it is proposed that the reverse Sellick’s maneuver appears to be best in terms of success rate and lowest number of adverse effects in the anesthetized, intubated patient and may be considered rather than blindly inserting the NG tube [9].

Confirmation of the proper placement of the NG tube is another topic that has been studied. Methods include radiography, physical signs of respiratory distress, aspirate appearance, ultrasonography, aspirate pH testing, carbon dioxide detection, and auscultation [12–16]. Given the risk of false positive results with the majority of these techniques, radiographic documentation remains the preferred technique, especially in the pediatric-aged patient. With the advent and increased use of POCUS, it is likely that bedside gastric ultrasonography may offer a rapid, bedside technique to identify the intragastric location of the tip of the NG tube without avoiding ionizing radiation.

### Learning points

Our two patients contribute to the growing literature of knotted and misplaced NG tubes by demonstrating an uncommon, but clinically significant complication in tracheally intubated, anesthetized pediatric patient. Both cases highlight that despite what appears to be uneventful placement, NG tube knotting and misplacement may occur. These complications including knotting may lead to safety concerns including inadvertent endotracheal extubation or resistance to withdrawal during NG tube manipulation. These cases reinforce that there may be a higher risk of complications in tracheally intubated and/or anesthetized patients. Evidence-based medicine suggests that successful placement may be facilitated by use of the reverse Sellick’s maneuver. Regardless of the technique used, radiographic confirmation is suggested prior to the administration of medications or enteral nutrition.

## Data Availability

Any inquiries regarding supporting data availability of this study should be directed to the corresponding author.
